# Cognitive Outcomes in Myelin Oligodendrocyte Glycoprotein‐IgG Associated Disease Compared to Multiple Sclerosis

**DOI:** 10.1002/brb3.70310

**Published:** 2025-02-28

**Authors:** Eileen Galvani, Jeffrey A. Cohen, John T. Martin, Kunio Nakamura, Amy Kunchok, Rachel Galioto

**Affiliations:** ^1^ Case Western Reserve University School of Medicine Cleveland Ohio USA; ^2^ Mellen Center for Multiple Sclerosis Neurological Institute Cleveland Clinic Cleveland Ohio USA; ^3^ Department of Psychological Sciences Kent State University Kent Ohio USA; ^4^ Department of Biomedical Engineering Cleveland Clinic Cleveland Ohio USA

**Keywords:** cognitive impairment, MOGAD, multiple sclerosis, Myelin oligodendrocyte glycoprotein‐IgG associated disease, neuropsychological test

## Abstract

**Background:**

Little is known about cognitive outcomes in people with myelin oligodendrocyte glycoprotein‐IgG associated disease (pwMOGAD). While there are similarities between MOGAD and multiple sclerosis (MS), further evaluation is needed to establish the distinct cognitive trajectories for each disease.

**Methods:**

Retrospective review of subjective cognitive changes was performed for adult pwMOGAD. Subsets of pwMOGAD completed a cognitive screening measure and/or comprehensive neuropsychological testing. Their performance was compared to age, sex, and race matched people with MS (pwMS; 3:1 ratio).

**Results:**

Approximately half (51.6%) of pwMOGAD (*n* = 63) endorsed cognitive dysfunction. On a cognitive screening measure (Processing Speed Test [PST]), performance did not statistically differ between pwMOGAD (*n* = 36; *M* = −0.06, SD = 1.36) and pwMS (*n* = 108; *M* = −0.04, SD = 1.30), *t*(62.35) = 0.18, *p* = 0.86. Of the pwMOGAD who completed neuropsychological testing (total *n* = 14), 57% demonstrated cognitive impairment on at least one test, and 36% demonstrated impairment on two or more tests. These rates did not differ from pwMS. In pwMOGAD, deficits were most common in processing speed (38.5%) and verbal fluency, spatial judgment, and immediate list recall (14.3% each). PwMOGAD performed better than pwMS on measures of visuomotor processing speed (*β* = −0.378, *t* = −2.68, *p* = 0.01) and cognitive flexibility (*β* = −0.33, *t* = −2.27, *p* = 0.03) after controlling for age, sex, race, education, and disease duration.

**Conclusions:**

Subjective cognitive complaints and objective cognitive deficits were common among pwMOGAD. Approximately one‐third of pwMOGAD demonstrated clinically significant cognitive impairment (2+ tests impaired) and individual level deficits were most common on measures of processing speed, verbal fluency, spatial judgment, and word list recall.

## Graphical abstract

Cognitive complaints were common among people with MOGAD (pwMOGAD; 54%) though only 8.3% screened positive for cognitive deficits on the Processing Speed Test (compared to 13.9% of pwMS). Minimal differences on neuropsychological tests were seen between people with pwMOGAD and pwMS, though pwMOGAD performed better on Trail Making Test Part A and B and delayed free recall of a word list.

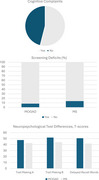

## 1 Introduction

Myelin oligodendrocyte glycoprotein antibody‐associated disease (MOGAD) is a central nervous system disorder with immune‐mediated demyelination. Clinical manifestations typically include optic neuritis (ON), transverse myelitis (TM), brainstem syndromes, and encephalitis, or a combination of these features (e.g., encephalomyelitis) (Banwell et al. 2023). The distribution of clinical phenotypes varies by age with isolated ON being more common in adults and acute disseminated encephalomyelitis (ADEM) being more common in children. In addition, adults are more likely than children to have a relapsing course and frequently recurrent ON (Cobo‐Calvo et al. 2021). Distinct from multiple sclerosis (MS), people with MOGAD (pwMOGAD) have lower brain lesion burden, and brain lesions that occur in MOGAD have different temporal dynamics to MS and can recede on subsequent imaging (Jarius et al. 2016; Cacciaguerra et al. 2023; Redenbaugh et al. 2023). Whether neurodegeneration is a feature of MOGAD is not yet well‐understood.

In contrast to MS, which is known to have a neurodegenerative component and a high proportion of patients experiencing cognitive impairment, associated with chronic inflammation and lesion burden, (Achiron et al. 2013; Olazarán et al. 2009; Preziosa et al. 2024) little is known about cognitive manifestations in pwMOGAD. A few studies have described cognitive deficits, involving processing speed, executive function, and visual‐motor skills, and school problems among pediatric patients with relapsing MOGAD (Hacohen et al. 2018; Zhou et al. 2019; Fabri et al. 2022; Deiva et al. 2020; Tan et al. 2021). Unsurprisingly, cognitive problems appear more commonly among pediatric patients with extensive brain lesions (Zhou et al. 2019) and those who present with acute or multiphasic encephalomyelitis (ADEM/MDEM) as opposed to ON or TM phenotypes (Olazarán et al. 2009; Deiva et al. 2020).

Adult pwMOGAD have demonstrated greater cortical and subcortical gray matter atrophy and lower scores on measures of global cognition, verbal memory, and processing speed compared to controls (Zhuo et al. 2021). A recent study showed that 7 of 32 pwMOGAD showed cognitive deficits, most commonly in mental flexibility, attention, and verbal working memory (Kogel et al. 2024). Finally, in a study of 113 pwMOGAD, 11% of patients showed impairment in two or more cognitive measures with the most common deficits being in processing speed and semantic fluency (Passoke et al. 2024). In pooled cohorts of adult and pediatric pwMOGAD, 5%–8% were noted to exhibit memory and/or concentration problems, but cognition was not systematically assessed via neuropsychological assessment (Jurynczyk et al. 2017; Netravathi et al. 2021). In a recent study of nine pwMOGAD nearly half of the sample (44%) demonstrated impairment in at least one cognitive domain (Li et al. 2023).

MOGAD is distinct from MS in that patients tend to have better long‐term clinical outcomes in terms of resolution of lesions on repeat MRI, visual acuity and disability measured by the expanded disability status score (EDSS) (Sechi et al. 2021; Lopez‐Chiriboga et al. 2020). Studies in patients with MS (pwMS) have demonstrated that cognitive dysfunction is common, particularly involving slowed processing speed, learning/memory difficulties, and executive dysfunction, which is associated with decreased cortical gray matter volume and higher mean T2 hyperintense lesion volume (De Meo et al. 2021). Few studies have comprehensively described cognitive outcomes in pwMOGAD or compared cognitive outcomes between MOGAD and MS.

The aim of this study is to describe cognitive outcomes in pwMOGAD, including subjective report and performance on cognitive screening measures and comprehensive neuropsychological testing. Performance on objective measures was also compared to a matched sample of pwMS. We hypothesized that cognitive complaints would be common in pwMOGAD, though pwMOGAD were expected to perform relatively better on neuropsychological testing than pwMS due to the paucity of chronic brain lesions in the majority of patients (Sechi et al. 2021; Lopez‐Chiriboga et al. 2020).

## 2 Methods

### 2.1 Participants and Procedures

Adult pwMOGAD were recruited from the Mellen Center for Multiple Sclerosis at Cleveland Clinic. All were MOG‐IgG positive in serum (Mayo Clinic Laboratories, Associated Regional and University Pathologists, Inc.) and fulfilled diagnostic criteria for MOGAD (Banwell et al. 2023).

Cognitive screening and neuropsychological test performance was compared between pwMOGAD and pwMS who were derived from existing IRB‐approved clinical neuropsychological registries. Matched sample size was 1 pwMOGAD:3 pwMS. The matching procedure was blind to cognitive screening and/or neuropsychological test performance and considered the best match from the available database based on age, sex, race, and disease duration. All pwMS fulfilled 2017 McDonald diagnostic criteria (Thompson et al. 2018). Only patients with relapsing‐remitting MS were included. Inclusion criteria for both groups were age ≥ 18 years, no history of neurologic disorder other than MOGAD or MS and being primarily English‐speaking. Complete NPT data was required for the MS group.

All procedures were performed in compliance with relevant laws and institutional guidelines. The study conforms with the World Medical Association Declaration of Helsinki. This study was approved by the Cleveland Clinic Institutional Review Board. Informed consent was obtained for all subjects.

### 2.2 Cognitive Complaints

Chart review was conducted to determine the presence of cognitive complaints, as defined by complaints from the patient recorded at any clinical visit following their MOGAD diagnosis.

### 2.3 Cognitive Screening

A subset of pwMOGAD and a matched sample of pwMS completed the Processing Speed Test (PST; Rao et al. 2017), a cognitive screening tool modeled after the Symbol Digit Modalities Test (SDMT; Smith 1982) administered during routine clinical care between November 2015 and March 2023.

### 2.4 Neuropsychological Testing

A subset of pwMOGAD and a matched sample of pwMS underwent comprehensive neuropsychological testing between February 2017 and December 2023. The test battery included measures of processing speed (SDMT; Smith 1982), Paced Auditory Serial Addition Test (PASAT; Gronwall 1977), Trail Making Test Part A (TMTA; Heaton et al. 2004), executive function (Wisconsin Card Sorting Test [WCST; Heaton et al. 1993], Trail Making Test Part B [TMTB; Heaton et al. 2004]), language (Boston Naming Test [BNT; Heaton et al. 2004], Controlled Oral Word Association Test [COWAT; Heaton et al. 2004]), and memory (California Verbal Learning Test–Second Edition [CVLT‐II; Delis et al. 1987], Brief Visuospatial Memory Test‐Revised (BVMT‐R) (Benedict 2023).

### 2.5 Brain MRI

Brain MRI was obtained as part of routine clinical care. The presence or absence of T2 lesions on brain MRI closest to cognitive evaluation in the MOGAD sample was based on the radiology report.

### 2.6 Statistical Analyses

Neuropsychological test scores were normalized using published normative data. *T* scores were truncated at ±3 standard deviations from the mean. Impairment rates were calculated for each test. Descriptive statistics, independent samples *t* tests (age, education), Mann–Whitney *U* (disease duration), and chi‐square analyses (sex, race) were used to characterize the sample, and compare the MOGAD and MS groups on demographic and clinical variables. Bivariate correlation analyses examined the association between neuropsychological test performance and disease duration. Chi‐square analyses examined group differences in impairment rates (≥ 1.5 standard deviations below the normative mean) on the PST and neuropsychological tests. Independent samples *t* tests examined group differences in test performance. Follow‐up linear regression analyses were conducted for any test with significant findings to examine the impact of group (MOGAD, MS) on PST scores and neuropsychological test performances after controlling for age, education, sex, race (White/non‐White), and disease duration. Analyses were conducted using SPSS v28.

## 3 Results

### 3.1 Cognitive Complaints

A total of 63 pwMOGAD were identified, of whom 62% were female, averaging 36.0 ± 14.0 years of age. Median MOG titer was 1:100 (range 1:20–1:1000). The majority (66.7%) had a monophasic course and 39.7% (*n* = 25) had brain lesions. The most common initial phenotype was ON (47.6%) followed by TM (30.2%). Additional phenotypes included ADEM (6.3%), brainstem (3.2%), and multifocal cortical lesions (1.6%) and 11.1% presented with a combination of symptoms.

Fifty‐four percent (*n* = 34) of pwMOGAD reported cognitive symptoms at some point following their diagnosis. There was no significant difference in sex distribution (*χ* ^2^ (*N* = 63) = 1.03, *p* = 0.38, *p* = 0.031) or age (*t*(61) = −0.44, *p* = 0.66) between patients with and without cognitive complaints. Cognitive complaints were more common among those with a relapsing course (*χ* ^2^ (*N* = 63) = 3.87, *p* = 0.049) but rates of cognitive complaints did not differ between those with and without brain lesions (*χ* ^2^ (*N* = 63) = 1.68, *p* = 0.20).

### 3.2 Cognitive Screening With PST

Thirty‐six pwMOGAD completed the PST and were age, sex, and race matched to 108 pwMS with PST. There were no significant differences in sex (72.2% female in both groups), race (77.8% vs. 80.6%, *p* = 0.324), age (36.6 ± 11.5 vs. 36.4 ± 11.2 years, *p* = 0.938), years of education (15.0 ± 2.3 vs. 15.2 ± 2.2 years, *p* = 0.778), or disease duration (5.7 ± 9.8 vs. 4.4 ± 3.5 years, *p* = 0.221). Twenty pwMOGAD (56%) had brain lesions. Table 1 provides characteristics of the sample.

**TABLE 1:** Sample characteristics.

	Cognitive screening			Neuropsychological testing		
Clinical variables	MOGAD (*n* = 36)	MS (*n* = 108)	*p*	MOGAD (*n* = 14)	MS (*n* = 42)	*P*
Age, years, mean (SD)	36.6 (11.5)	36.4 (11.2)	0.94	42.5 (13.4)	42.6 (12.0)	0.98
Female, *n* (%)	26 (72.2)	78 (72.2)	1.00	8 (57.1)	32 (76.2)	0.17
White, *n* (%)	28 (77.8)	87 (80.6)	0.72	12 (85.7)	36 (85.7)	1.00
Education, years, mean (SD)	15.0 (2.3)	15.2 (2.2)	0.78	15.4 (2.8)	15.2 (2.4)	0.08
Disease duration, years, median	5.7 (9.8)	4.4 (3.5)	0.22	2.0	4.5	0.05
MOG titer, median	1:100^a^	—	—	1:100	—	—
Brain lesions, *n* (%)	20 (55.6%)	—	—	5 (35.7%)	—	—

Abbreviations: MOGAD, myelin oligodendrocyte glycoprotein antibody‐associated disease; MS, multiple sclerosis.

^a^ Missing data for two patients.

Impairment on PST was observed in 8.3% of pwMOGAD and 13.9% of pwMS, a difference which was not statistically significant, *χ* ^2^ (*N* = 144) = 0.76, *p* = 0.38. Mean performance fell in the average range for both groups (MOGAD *M* = −0.06, SD = 1.36; pwMS *M* = −0.04, SD = 1.30), and did not significantly differ between groups, *t*(62.35) = 0.18, *p* = 0.86.

### 3.3 Neuropsychological Testing

Fourteen pwMOGAD completed neuropsychological testing. These patients averaged 42.50 ± 13.34 years of age, had 15.43 ± 2.79 years of education, were mostly White (85.7%) and approximately half (57.1%) were female. Median disease duration was 2.00 years (range = 0–26). The most common clinical phenotype was ON (64.3%), followed by TM/ON (21.4%), and 64.3% had a relapsing course see Table 1.

These pwMOGAD were matched to 42 age, sex, and race pwMS who completed neuropsychological testing. As expected due to matching procedures, there were no significant differences between groups in sex (*p* = 0.17), race (*p* = 0.83), age (*p* = 0.98), years of education (*p* = 0.78). The difference in disease duration neared significance (*p* = 0.05).

Among pwMOGAD, mean scores fell within the average range across all neuropsychological tests. However, 57.1% (*n* = 8) of pwMOGAD demonstrated impairment on at least one test, with deficits being most common on measures of processing speed (PASAT; 38.5%), verbal fluency (COWAT; 14.3%), spatial judgment (JOLO; 14.3%), and immediate list recall (14.3%). Approximately one‐third (35.7%; *n* = 5) of pwMOGAD demonstrated impairment on 2+ tests. Among these patients, all presented initially with ON, one of whom also presented with TM. Three of the five had a monophasic course and did not show brain lesions on brain MRI. Two of the five had a relapsing course (one with three attacks over 6 years, one with two attacks over 5 years) both of whom showed brain lesions on brain MRI. Disease duration was not significantly associated with performance on any of the neuropsychological tests (*p*'s ranged from 0.27 to 0.91). Characteristics and neuropsychological test performance for all pwMOGAD can be found the Supporting Information.

Compared to pwMS, pwMOGAD performed significantly better in terms of mean performance on measures of processing speed (Trails A, *t*(42) = 2.78, *p* < 0.01), cognitive flexibility (Trails B, *t*(42) = 2.44, *p* = 0.019), and delayed recall of a word list (*t*(54) = 2.20, *p* = 0.03). No other differences between groups emerged on neuropsychological tests see Table 2.

**TABLE 2:** Independent samples *t* tests comparing performance on neuropsychological tests of the MOGAD and MS samples.

Neuropsychological tests^a^	MOGAD (*n* = 14)		MS (*n* = 42)			
	*n*	Mean (SD)	*n*	Mean (SD)	*t*	*p*
PASAT	13	42.5 (13.6)	39	42.7	−0.03	0.98
SDMT	14	47.0 (10.6)	42	44.0 (12.3)	0.82	0.42
TMTA	11	47.7 (14.4)	33	42.6 (10.1)	2.78	0.008
TMTB	11	51.8 (15.1)	33	42.1 (10.1)	2.44	0.02
WCST total errors	13	48.2 (8.1)	39	44.2 (9.3)	1.41	0.17
COWAT	14	45.9 (10.6)	42	43.9 (10.3)	0.62	0.54
BNT	14	40.8 (4.4)	42	43.1 (10.1)	−1.19	0.24
JOLO	14	48.6 (8.9)	42	50.4 (8.4)	−0.71	0.48
CVLT immediate	14	50.4 (10.2)	42	45.6 (10.2)	1.53	0.13
CVLT delayed	14	50.7 (10.7)	42	41.8 (13.8)	2.20	0.03
BVMT‐R immediate	14	51.4 (8.6)	42	48.1 (12.9)	0.89	0.38
BVMT‐R delayed	14	52.9 (8.5)	42	48.6 (13.9)	1.36	0.18

Abbreviations: BNT, Boston Naming Test; BVMT‐R, Brief Visuospatial Memory Test‐Revised; COWAT, Controlled Oral Word Association Test; CVLT2, California Verbal Learning Test; JOLO, Judgment of Line Orientation; PASAT, Paced Auditory Serial Addition Test; SDMT, Symbol Digit Modalities Test; TMTA, Trail Making Test Part A; TMTB, Trail Making Test Part B; WCST, Wisconsin Card Sorting Test.

^a^ Neuropsychological test scores are presented as *T* scores (mean = 50, standard deviation = 10).

After controlling for age, education, sex, race, and disease duration, group remained a significant predictor of Trails A (*β* = −0.378, *t* = −2.68, *p* = 0.01) and Trails B (*β* = −0.33, *t* = −2.27, *p* = 0.03) performance but not delayed word recall (*β* = −0.22, *t* = −1.74, *p* = 0.09).

Similar rates of one (64.3% MOGAD vs. 81% MS, *χ* ^2^ (*N* = 56) = 1.64, *p* = 0.20, Cramer's *V* = 0.17) and two (35.7% MOGAD vs. 64.3% MS, *χ* ^2^ (*N* = 56) = 3.50, *p* = 0.06, Cramer's *V* = 0.25) test impairments were seen between patient groups.

## 4 Discussion

This study described cognitive outcomes in pwMOGAD, including subjective cognitive complaints and performance on both cognitive screening and comprehensive neuropsychological testing in a sample of pwMOGAD. Subjectively, approximately half (54%) of the pwMOGAD in this study reported cognitive symptoms. This is consistent with results of formal neuropsychological testing which found objective deficits on at least one test in 57% of the pwMOGAD sample. This rate is similar to what was recently described by Li et al. (2023) who found impairment on at least one test in 44% of their sample. Clinically significant cognitive impairment (2+ tests impaired) was found in greater than one‐third (35.7%) of pwMOGAD. Thus, this study found that subjective cognitive complaints and objective cognitive deficits on neuropsychological testing are relatively common among pwMOGAD. However, the mechanisms underlying cognitive dysfunction in this group remains unclear. Specifically, most cognitively impaired pwMOGAD had minimal lesion burden on brain MRI and the majority had a monophasic course. These results suggest that cognitive changes could be related to cerebral pathology that is not evident on conventional MRI (microstructural or functional changes), or contributed to by other clinical factors, such as psychiatric conditions, sleep problems, and/or medications such as corticosteroids.

Interestingly, impairment on a screening measure assessing processing speed was less common, observed in only 8.3% of the MOGAD sample, suggesting that brief screening measures may not be adequate to detect the range of cognitive problems seen in pwMOGAD. We have raised similar criticisms of these screening measures when used in pwMS (Galioto et al. 2021).

In contrast to our hypothesis, this study did not reveal many differences between patients with MOGAD and MS. Specifically, no differences in cognitive screening test performance emerged between groups. On more detailed neuropsychological testing, pwMOGAD performed better on tests of psychomotor speed, cognitive flexibility, and delayed recall of a word list, though the latter finding did not withstand correction for matching variables. Similarly, rates of test impairment (defined as 1 or 2+ test impairment) did not differ between groups. There are several potential reasons why our hypothesis was not supported. First, the small sample size may have limited our ability to detect meaningful differences between groups. Future studies should therefore investigate potential differences between patients with MOGAD and MS in larger samples. In addition, this study included patients who were relatively early in the disease course. Though it is well recognized that there is a neurodegenerative component to MS, whether this is the case for MOGAD is unknown. Thus, the two groups may show greater divergence on cognitive test performance at a later timepoint, highlighting the need for longitudinal studies.

When considering cognitive impairment among PwMOGAD, the impact of any residual visual disturbance may be important to consider as it could influence performance on some tasks. However, in this study it is unlikely that visual dysfunction greatly influenced findings as the three of the four most commonly impaired tests in our sample had no visual demands. Regarding cognitive complaints in the full sample, disease duration at the time of complaint was not collected. Therefore, we could not determine whether the presence of cognitive complaints varies throughout the course of the disease. This is an important question that should be addressed in future studies. There are a few additional limitations of this study to consider. First, selection bias could have influenced these results as it may have impacted patients' decision to participate in the study. Specifically, patients with subjective cognitive complaints may have been more likely to participate than those without. Second, due to the retrospective and observational nature of this study, the timing of neuropsychological testing was not standardized. Finally, administrative error led to some patients missing the Trail Making Test Parts A or B, which may underestimate rates of impairment on this test.

Despite these limitations, this study has systematically assessed multiple domains of cognition in adults pwMOGAD. Comparison of these results with well‐established data on cognitive outcomes in MS is important in differentiating these two diseases in terms of disease course and outcomes. Future studies examining changes in each domain of cognition over time would be useful in developing prognostics for pwMOGAD and tracking functional outcomes.

## Author Contributions

**Eileen Galvani**: conceptualization, funding acquisition, writing–original draft, formal analysis. **Jeffrey A. Cohen**: writing–review and editing. **John T. Martin**: writing–review and editing, project administration. **Kunio Nakamura**: writing–review and editing. **Amy Kunchok**: conceptualization, funding acquisition, writing–review and editing, methodology, supervision, resources. **Rachel Galioto**: Supervision, formal analysis, writing–original draft, writing–review and editing, conceptualization, methodology.

## Conflicts of Interest

Kunio Nakamura received personal compensation for licensing fee from Biogen and scientific advisory meeting from INmune Bio. Jeffrey A. Cohen reports personal compensation for consulting for Astoria, Bristol‐Myers Squibb, Convelo, EMD Serono, FiND Therapeutics, INMune, and Sandoz; and serving as an Editor of *Multiple Sclerosis Journal*. The other authors declare no conflicts of interest.

### Peer Review

The peer review history for this article is available at https://publons.com/publon/10.1002/brb3.70310.

## Supporting information



Supporting Information

## Data Availability

The data that support the findings of this study are available from the corresponding author upon reasonable request.
