# Supplementary material for: Cognitive Outcomes in Myelin Oligodendrocyte Glycoprotein‐IgG Associated Disease Compared to Multiple Sclerosis
Source: Brain Behav. 2025 Feb 28;15(3):e70310. doi: 10.1002/brb3.70310 (PMC11870788; doi:10.1002/brb3.70310)
Supplement: Supplementary file 1 — Supporting Information [file BRB3-15-e70310-s001.docx]

**Supporting Information**

**MOGAD Sample Characteristics and Neuropsychological Test Performance**

| Patient | 1 | 2 | 3 | 4 | 5 | 6 | 7 | | 8 | 9 | 10 | 11 | 12 | 13 | 14 |
| --- | --- | --- | --- | --- | --- | --- | --- | --- | --- | --- | --- | --- | --- | --- | --- |
| Age, years | 37 | 48 | 39 | 44 | 24 | 55 | 51 | | 29 | 52 | 33 | 28 | 33 | 50 | 32 |
| Sex | Female | Female | Male | Female | Female | Male | Male | | Male | Female | Female | Male | Female | Male | Female |
| Race | White | White | White | White | Black | White | White | | White | Black | White | White | White | White | White |
| Education, years | 16 | 19 | 17 | 14 | 14 | 16 | 8 | | 16 | 18 | 16 | 16 | 18 | 12 | 16 |
| Relevant comorbidities | Depression | T1DM, Depression | None | Hashimoto’s disease | PTSD, somatic symptom disorder, functional movement disorder | HTN, HLD | None | | T2DM | Depression | Obesity | Anxiety, depression | HLD, HTN, T2DM, rheumatoid arthritis | HTN | None |
| Relapsing Course | Yes | Yes | No | Yes | Yes | No | Yes | | No | No | No | Yes | Yes | Yes | Yes |
| Phenotype | TM, ON | TM, ON | ON | ON | ON | ON | TM, ON | | ADEM | ON | ON | ON | Multifocal brain lesions | ON | ON |
| OCBs | No | No | No | No | No | No | No | | No | No | No | No | No | No | No |
| Duration, years | 4 | 6 | 5 | 9 | 1 | 1 | 26 | | <1 | 3 | 1 | 1 | <1 | 1 | 4 |
| MOG Titer | - | 1:1000 | - | 1:20 | 1:100 | 1:100 | 1:100 | | 1:100 | 1:1000 | 1:100 | 1:1000 | 1:1000 | 1:100 | 1:100 |
| Brain lesions | + | + | - | - | - | - | + | | - | - | - | - | + | - | + |
| Brain MRI Impression | Lesion in the ventral left thalamus/genu  of the left internal capsule; mild degree of white matter  signal abnormality | Mild lesion burden. Moderate parenchymal volume loss | Normal | Normal | Normal | Normal | Punctate lesions of the  supratentorial subcortical white matter | | Gyriform  signal abnormality, swelling, and sulcal effacement, predominantly  of the left parietal lobe | Stable enlargement of the bilateral optic nerve sheathes. | Normal | Bilateral optic nerve enhancement | Bilateral enhancement of the left frontal operculum/corona radiata. | Normal | Multiple white matter lesions |
| Testing to MRI, months | 3.3 | 2.1 | 4.7 | 0.4 | 5.9 | 6.7 | 7.1 | | 0.4 | 30.0 | 6.0 | 2.8 | 4.0 | 0.4 | 24.0 |
| Neuropsychological Test Performance | | | | | | | | | | | | | |  |  |
| PASAT | 57 | 20 | 58 | 45 | 37 | 58 | n.d. | 33 | | 31 | 31 | 45 | 30 | 64 | 44 |
| TMTA | 66 | n.d. | n.d. | n.d. | 37 | 59 | 47 | 54 | | 34 | 39 | 58 | 46 | 57 | 45 |
| SDMT | 64 | 20 | 50 | 41 | 43 | 52 | 48 | 55 | | 43 | 39 | 49 | 47 | 61 | 46 |
| TMTB | 80 | n.d. | n.d. | n.d. | 53 | 56 | 52 | 47 | | 41 | 42 | 56 | 38 | 73 | 28 |
| WCST | 50 | 34 | 42 | 46 | 51 | 51 | 55 | 55 | | 47 | 38 | 56 | n.d. | 62 | 40 |
| COWAT | 61 | 65 | 29 | 44 | 45 | 51 | 53 | 36 | | 34 | 40 | 42 | 37 | 57 | 48 |
| BNT | 38 | 39 | 34 | 40 | 49 | 36 | 46 | 40 | | 42 | 36 | 40 | 47 | 40 | 44 |
| JOLO (%ile) | 86% | 40% | 72% | 4% | 22% | 72% | 40% | 56% | | 40% | 40% | 72% | 56% | 72% | 2% |
| CVLT IR | 49 | 55 | 49 | 55 | 59 | 56 | 56 | 58 | | 42 | 34 | 43 | 52 | 68 | 30 |
| CVLT DR | 40 | 55 | 50 | 55 | 65 | 50 | 55 | 60 | | 50 | 25 | 45 | 55 | 65 | 40 |
| BVMT IR | 62 | 49 | 54 | 44 | 43 | 57 | 56 | 62 | | 60 | 48 | 49 | 49 | 56 | 31 |
| BVMT DR | 63 | 54 | 40 | 53 | 53 | 61 | 65 | 61 | | 50 | 49 | 48 | 46 | 60 | 37 |

Note: Test performances are T-scores (mean=50, sd=10) unless otherwise noted; Grey cells indicate impaired scores (≥ 1.5 standard

Abbreviations: T1DM = Type 1 diabetes mellitus; T2DM = Type 2 diabetes mellitus; HTN = hypertension; HLD = hyperlipidemia; PTSD = posttraumatic stress disorder; n.d. = not done; PASAT=Paced Auditory Serial Addition Test; TMTA=Trail Making Test – Part A; SDMT=Symbol Digit Modalities Test; TMTB = Trail Making Test- Part B; WCST=Wisconsin Card Sorting Test; COWAT=Controlled Oral Word Association Test; BNT=Boston Naming Test; JOLO=Judgment of Line Orientation; CVLT2=California Verbal Learning Test; IR = Immediate Recall, DR = Delayed Recall; BVMT-R = Brief Visuospatial Memory Test-Revised
